# Incorporating ultrasound training into undergraduate medical education in a faculty-limited setting

**DOI:** 10.1186/s12909-023-04227-y

**Published:** 2023-04-19

**Authors:** Kimberly M. Rathbun, Arjun N. Patel, Jacob R. Jackowski, Matthew T. Parrish, Ryan M. Hatfield, Tyler E. Powell

**Affiliations:** 1 Department of Emergency Medicine, Augusta University, University of Georgia Medical Partnership, Athens, GA Greece; 2 Department of Surgery, Medical University of South Carolina, Charleston, SC USA; 3 Department of Orthopedic Surgery, Mayo Clinic, Rochester, MN USA; 4 Department of General Surgery, Mayo Clinic, Rochester, MN USA; 5 Department of Emergency Medicine, Prisma Health, Columbia, SC USA; 6 Department of Anesthesia and Perioperative Medicine, Mayo Clinic, Rochester, MN USA

**Keywords:** POCUS, Ultrasound Education, Point of Care Ultrasound, Undergraduate Medical Education

## Abstract

**Background:**

Point of care ultrasound (POCUS) is becoming a major extension of patient care. From diagnostic efficacy to its widespread accessibility, POCUS has expanded beyond emergency departments to be a tool utilized by many specialties. With the expansion of its use, medical education has begun to implement ultrasound education earlier in curricula. However, at institutions without a formal ultrasound fellowship or curriculum, these students lack the fundamental knowledge of ultrasound. At our institution, we set out to incorporate an ultrasound curriculum, into undergraduate medical education utilizing a single faculty member and minimal curricular time.

**Methods:**

Our stepwise implementation began with the development of a 3-hour fourth-year (M4) Emergency Medicine clerkship ultrasound teaching session, which included pre- and post-tests as well as a survey. The success with this session progressed to the development of a designated fourth-year ultrasound elective, which was evaluated with narrative feedback. Finally, we developed six 1-hour ultrasound sessions that correlated with first-year (M1) gross anatomy and physiology. A single faculty member was responsible for this curriculum and other instructors included residents, M4 students, and second-year (M2) near-peer tutors. These sessions also included pre- and post-tests and a survey. Due to curricular time limitations, all but the M4 Emergency Medicine clerkship session were optional.

**Results:**

87 students participated in the emergency medicine clerkship ultrasound session and 166 M1 students participated in the voluntary anatomy and physiology ultrasound sessions. All participants agreed or strongly agreed that they would like more ultrasound training, that ultrasound training should be integrated into all four years of undergraduate medical education. Students were in strong agreement that the ultrasound sessions helped increase understanding of anatomy and anatomical identification with ultrasound.

**Conclusion:**

We describe the stepwise addition of ultrasound into the undergraduate medical education curriculum of an institution with limited faculty and curricular time.

**Supplementary Information:**

The online version contains supplementary material available at 10.1186/s12909-023-04227-y.

## Background

Point of care ultrasound (POCUS) is becoming increasingly common in medical practice across a variety of specialties. As a brief, targeted, and clinician-directed imaging study performed at the patient’s bedside, POCUS is favored for its accessibility and ease of use as a diagnostic tool allowing the clinician to rapidly answer specific clinical questions, optimize patient care, and expedite management [1]. First becoming widely utilized in the emergency department for various patient presentations such as trauma, undifferentiated shock, and acute abdominal pain, POCUS use has now spread to many other specialties [2–5]. In addition to guiding clinical decision-making, ultrasound is playing an increasingly important role in medical education. In 2012, the Accreditation Council for Graduate Medical Education (ACGME) and the American Board of Emergency Medicine (ABEM) included ultrasound as one of the milestones for emergency medicine residents with expansion into other specialties training programs [6–9]. The utility of incorporating ultrasound training at both the residency and fellowship level suggested that trainees could benefit from even earlier exposure [10–11].

Medical schools have responded to these trends by incorporating ultrasound into undergraduate medical education with the breadth of these programs varying from fully integrated, four-year programs to limited integration of ultrasound into preexisting courses [12– 19]. Studies indicate that incorporating imaging-based curricula into undergraduate medical education helps students to better understand disease processes and diagnosis [20–22]. In addition to supplementing preexisting learning objectives, exposure to an ultrasound curriculum as a medical student impacts individual preparedness to begin using ultrasound diagnostically in residency [23].

Recommended curricula and educational strategies for medical schools have been previously published in Europe and the United States; however, a 2012 survey of US medical schools showed that only 62% of survey participants reported focused ultrasound education in their UME curricula (Beeson 2013) with a subsequent survey from 2014 indicating that only 27.7% of schools had a formal curriculum [17, 24−27]. By 2019, that percentage had risen to 74% [28]. Approximately 50% of Canadian medical schools had implemented focused ultrasound education in a 2014 survey [29]. These studies note that major barriers to the implementation of ultrasound education are curricular time, faculty time and expertise, and access to equipment.

At our institution, medical students were exposed to ultrasound intermittently during clinical rotations; however, there was no formal ultrasound instruction within our curriculum. We sought to take steps to introduce ultrasound instruction in the undergraduate medical curriculum using a single faculty member and minimal curricular time. Our stepwise implementation included the development of an fourth-year (M4) Emergency Medicine clerkship session, a designated M4 ultrasound elective, and an adjunct ultrasound curriculum correlating with first-year (M1) gross anatomy and physiology.

## Methods

The Brody School of Medicine at East Carolina University, United States, admits 90 students per year and has a traditional four-year medical school (M1-M4) curriculum. The medical school is associated with a 974-bed tertiary-care teaching hospital that supports many residency and fellowship training programs. There is an emergency medicine residency, but no emergency ultrasound fellowship. The emergency medicine residency curriculum includes an ultrasound rotation during the intern year.

### Phase I: emergency medicine clerkship academic half day

Because the Emergency Medicine clerkship is required for all students and ultrasound is covered on the clerkship shelf exam, it was selected as the initial target for introductory medical student ultrasound education. In coordination with the emergency medicine clerkship director and executive curriculum committee, a 3-hour block of educational time was designated for ultrasound education. The sessions were held in the simulation center which provided lecture space with equipment, ultrasound machines with appropriate probes, and standardized patients. The sessions began with a 30-minute presentation by an Ultrasound faculty member discussing basic machine operation and the physics of ultrasound. Then, students viewed instructional videos made by the Society of Academic Emergency Medicine (SAEM) Academy of Emergency Ultrasound (AEUS) Narrated Lecture Series covering the extended focused assessment with sonography for trauma (eFAST), biliary ultrasound, and aortic ultrasound. Each video was followed by hands-on practice of that ultrasound examination on a standardized patient. Instructors included emergency medicine faculty and residents. The maximum number of students per group was seven. If necessary, the students were split into two groups for the hands-on practice. The students each completed a seven-item, five-point Likert Scale survey evaluating the effectiveness of the session. They also completed a pre-test and post-test that consisted of multiple-choice questions assessing ultrasound knowledge related to the session subject material and a free-response section assessing the ability to identify anatomical structures on ultrasound images.

### Phase II: emergency ultrasound elective

Once the emergency medicine clerkship ultrasound session was established and running smoothly, the creation of a four-week fourth-year medical student ultrasound elective was launched as the second phase of the project. The goal of this elective was to expose fourth-year medical students to the core ultrasound applications used in daily practice by emergency medicine physicians. This elective allowed students to develop hands-on skills in image acquisition and bedside interpretation of images, improving competency in Focused Assessment with Sonography for Trauma (FAST), Abdominal Aorta, Cardiac, Biliary, Renal, Thoracic, and Soft tissue/Abscess imaging. Students were also exposed to more advanced emergency ultrasound applications including ocular, advanced cardiac, deep vein thrombosis, testicular, and procedural applications. Course requirements included online didactics and 14 scanning shifts in the Emergency Department. Enrollment in the elective was limited to 10 students per academic year due to the faculty time required to complete the hands-on component. Preference was given to students planning to pursue residency in Emergency Medicine, Surgery, Radiology, or other ultrasound-intensive specialty. This course was evaluated by narrative feedback from students at the end of the academic year.

### Phase III: M1 anatomy and physiology correlation using ultrasound

The final phase of ultrasound integration was the creation of an ultrasound curriculum that correlated with the M1 anatomy and physiology courses. This ultrasound series consisted of 6 separate sessions: cardiac, aorta, upper abdominal, renal, pelvic, and ocular. The sessions were scheduled immediately following coverage of corresponding material in M1 anatomy and physiology. Due to limited curricular time, these sessions were voluntary for students. The second-year medical student leaders coordinated the timing of the sessions and notified M1 students of upcoming sessions. The sessions were held in the simulation center, which provided lecture space with equipment and ultrasound machines with appropriate probes. Each session was designed to last no more than 1 h and consisted of an introductory lecture created by the faculty member followed by hands-on ultrasound practice. For the hands-on component of the session, group sizes were limited to 5 students to one instructor and students practiced scans on each other. The sessions were created and led by one emergency medicine faculty member. Instructors included emergency medicine interns on their ultrasound rotation, M4 students enrolled in the ultrasound elective, and M2 student leaders who were selected to facilitate the sessions. Mirroring the emergency medicine clerkship ultrasound session, pre-module and post-module assessments were used that consisted of two parts: a session-specific multiple-choice section assessing related ultrasound knowledge and a free-response section assessing ability to identify anatomical structures on ultrasound images. Additionally, the students completed a seven-item, five-point Likert Scale survey regarding the usefulness of the session and whether it should be incorporated into the curriculum.

## Results

### Phase I: emergency medicine clerkship academic half day

During one academic year, 87 students participated in the emergency medicine clerkship ultrasound session. The mean percent correct on the pre-tests and post-tests were 53.5% (median 58.3%) and 80.3% correct (median 83.3%), respectively (p < 0.01) (Table 1). Overall, students showed a significant increase in basic ultrasound knowledge. Of the 87 students, 67 (77.0%) indicated minimal prior ultrasound experience (none, observational only, or performed < 10 scans), 16 (18.4%) indicated that they had performed > 10 scans before this experience, and four (4.6%) did not answer the question (Table 2). Survey results indicated that students found the educational session relevant and useful (Table 3). All but one student agreed or strongly agreed that ultrasound training is both relevant to their level of training and was relevant to the fourth year Emergency Medicine rotation. All participants agreed or strongly agreed that they would like more ultrasound training, that ultrasound training should be integrated into all four years of undergraduate medical education, and that ultrasound training applies to their future careers as physicians.

**Table 1:** Average pre-and post-test scores for EM clerkship ultrasound session

	Pre-Test Percent Correct	Post-Test Percent Correct
EM Clerkship ultrasound session	53.5%	80.3%

**Table 2:** Prior ultrasound experience of EM clerkship ultrasound session participants

None	1
Observation only	28
Performed < 10 scans	38
Performed 10–50 scans	12
Performed > 50 scans	4
No response	4
**Total**	**87**

**Table 3:** EM clerkship ultrasound session survey score on 5-point Likert scale (1 = Strongly Disagree, 5 = Strongly Agree)

Survey Question	Score*
I enjoyed this course.	4.76
Ultrasound education is relevant to my level of training.	4.80
Ultrasound training is relevant to the M4 Emergency Medicine rotation.	4.87
As a result of this training, my knowledge of ultrasound improved.	4.84
I have gained a basic understanding of ultrasound physics.	4.68
I can operate an ultrasound machine at a basic level.	4.62
I can image the liver, spleen, pelvis, aorta, gallbladder, and heart at a basic level.	4.61
Ultrasound training increased my knowledge of the anatomy of the abdomen.	4.59
I would like more ultrasound training.	4.82
Ultrasound training should be integrated into all 4 years of undergraduate medical education.	4.72
Ultrasound training applies to my future career as a physician.	4.80

### Phase II: emergency ultrasound elective

The M4 Emergency Ultrasound elective was very popular and reached the maximum enrollment of 10 students every year, with at least 10 other students having to be turned away yearly. Based on narrative feedback collected at the end of the course, students found the elective to be very useful for their medical training. They enjoyed the hands-on component of the rotation the most, especially noting that there were only one or two students with an instructor. They found that the online didactic materials were useful, especially with regards to pathologic findings that are not often seen. While they enjoyed practicing ultrasound on patients, students noted that they might have benefitted from additional practice on standardized patients early in the elective.

### Phase III: M1 anatomy and physiology correlation using ultrasound

Over two academic years, 166 M1 students participated in the voluntary anatomy and physiology ultrasound sessions (cardiac: 43 students; aorta: 27 students; upper abdomen: 34 students; renal: 23 students; pelvis: 12 students; ocular: 27 students). Due to the COVID-19 pandemic, the pelvis ultrasound session was unable to be held during the second year. When comparing pre-module and post-module scores, students demonstrated a statistically significant (all p < 0.05) increase in the basic ultrasound anatomical knowledge assessed through the multiple-choice section (Table 4) as well as image identification on ultrasound imaging (Table 5). Survey data (Table 6) from all ultrasound curriculum sessions demonstrated that students thoroughly enjoyed the ultrasound sessions while finding them extremely beneficial to their education. Students were in strong agreement that the ultrasound sessions helped increase understanding of anatomy and anatomical identification with ultrasound. Most students agreed or strongly agreed that ultrasound training is relevant to their level of training, correlated well with the gross anatomy curriculum, and should be incorporated into the M1 curriculum.

**Table 4:** Average pre-and post-test scores for M1 session ultrasound knowledge questions

Session	Year One Average Percent Correct		Year Two Average Percent Correct	
	Pre-Test	Post-Test	Pre-Test	Post-Test
Cardiac	15.79%	89.47%	19.57%	80.43%
Aorta	50.0%	96.67%	39.58%	95.83%
Upper Abdomen	35.0%	85.0%	41.67%	75.0%
Renal	50.0%	90.90%	47.92%	91.66%
Pelvis	65.91%	100.0%	COVID	COVID
Ocular	15.91%	100.0%	26.56%	87.50%

**Table 5:** Average pre-and post-test scores for M1 session image-based anatomical questions

Session	Year One Average Percent Correct		Year Two Average Percent Correct	
	Pre-Test	Post-Test	Pre-Test	Post-Test
Cardiac	30.0%	94.74%	40.0%	90.87%
Aorta	40.0%	95.0%	42.71%	98.96
Upper Abdomen	7.20%	94.40%	17.78%	74.44%
Renal	32.95%	82.82%	16.66%	99.07%
Pelvis	39.77%	98.70%	COVID	COVID
Ocular	68.18%	91.10%	19.53%	86.66%

**Table 6:** Average M1 session survey score on 5-point Likert scale (1 = Strongly Disagree, 5 = Strongly Agree)

Survey Question	Cardiac (n = 43)	Aorta (n = 27)	Upper Abdomen (n = 34)	Renal (n = 23)	Pelvis (n = 12)	Ocular (n = 27)
I enjoyed this course.	4.90	4.87	4.88	4.92	4.92	4.87
Ultrasound education is relevant to my level of training.	4.81	4.87	4.83	4.96	4.92	4.54
The pre-session lecture helped with my understanding of ultrasound.	4.81	4.80	4.93	4.92	4.92	4.81
This session increased my understanding of normal anatomy.	4.76	4.80	4.83	4.96	4.92	4.77
This session helped me identify anatomical features.	4.88	4.83	4.90	4.96	4.92	4.81
This session was well correlated with the first-year anatomy curriculum.	4.90	4.90	4.98	4.96	4.92	4.58
This session should be incorporated into the first-year anatomy and physiology curricula.	4.76	4.83	4.80	4.96	4.92	4.54

## Discussion

In this educational intervention, we describe the stepwise integration of ultrasound into undergraduate medical education in an institution with limited faculty and curricular time. Point-of-care ultrasound, due to its effectiveness as a cost-friendly diagnostic tool, is becoming increasingly relevant in medical practice and medical education. There is extensive literature demonstrating the utility of incorporating ultrasound education early in residency and fellowship training, and that the assimilation early into the medical school curriculum was associated with higher scores on image interpretation tests [10–11, 23]. Even with these positive outcomes, many schools have not been able to incorporate ultrasound sessions into their curriculum due to limited faculty and monetary resources [17, 27] . This paper aims to provide a framework for institutions limited by faculty availability to incorporate ultrasound into the pre-clinical and clinical years, address the many barriers faced in implementation, and demonstrate that this curriculum improves student performance and knowledge of anatomic relationships [17].

The introduction of ultrasound to the undergraduate medical student curriculum was initially implemented as a three-hour session during the M4 Emergency Medicine clerkship. Similar incorporation of ultrasound at the M3/M4 level has been attempted in the past with only a few specifically targeting emergency medicine [30–32]. This session was relatively easy to implement. Given that it required only 3 h of curricular time and ultrasound questions are now appearing on standardized emergency medicine tests, it made sense to add ultrasound to the EM clerkship didactic sessions. The institutional requirement that all students complete the M4 EM rotation ensured that every medical student was exposed to ultrasound prior to graduation. The sessions could be held with a single instructor, but there would be more downtime for students as they wait their turn to practice hands-on scanning. If less curricular time is available, much of the didactics are available online and the session could be held in a flipped classroom format with only the hands-on scanning session being in person. A flipped classroom format could limit the time needed to about an hour, assuming there are enough instructors. Additionally, we used paid standardized patients for the hands-on scanning, but students could scan each other in the case that a similar resource is not available.

After the fourth-year EM clerkship ultrasound session was established, a 4-week EM ultrasound elective was created to provide a more in-depth ultrasound experience for interested M4 students. Similar courses have been established at other medical schools [33]–[34]. The didactic component of the elective is all online, requiring minimal faculty time after the initial development. The didactic material we used for this elective is the same as the didactic material used for the EM intern ultrasound rotation, resulting in almost no extra time for curricular development. If an institution doesn’t already have such a rotation in place, the SAEM AEUS Narrated Lecture Series offers modules covering all relevant ultrasound exams. They offer short quizzes associated with the videos, but more extensive quizzes can be made (as was done in our case). The significant limiting factor for this elective is the amount of faculty time needed for hands-on scanning with the students. This was mitigated by having the students’ hands-on scanning time coincide with the EM intern’s scanning time. Care was taken to ensure that no more than three learners were present at any time. Students were also invited to attend the M4 EM clerkship ultrasound sessions to practice scanning. Additionally, once the M1 ultrasound sessions were developed, M4 elective students attended those sessions to practice scanning as well as to serve as near-peer instructors.

Following the successful implementation of the fourth-year ultrasound elective and the growing demand for ultrasound education, we sought to incorporate ultrasound earlier in the undergraduate medical curriculum. Because of the technology’s ability to demonstrate structural relationships, anatomy courses have become the natural target for incorporating ultrasound into pre-clinical undergraduate medical education. Previous research shows that students who participate in similar sessions believed the sessions improved their understanding of human anatomy and these feelings were objectively supported by improved test scores following instruction [12, 29, 35]. In addition, faculty that supervised similar sessions were more likely to assign students higher subjective ratings on the student’s ability to perform ultrasound and identify anatomical structures following ultrasound instruction than students who did not receive instruction [36–37]. The need for faculty, was mitigated by using M2 near-peer tutors as instructors with the faculty instructor floating from station-to-station to assist as needed. Because of limited curricular time, the M1 sessions were optional for students and were held outside of dedicated class time. To incorporate these sessions into existing curricular time, we plan to hold these sessions in conjunction with anatomy and physiology laboratory time. Half of the students will participate in the laboratory and half will participate in the ultrasound session and then the groups will switch halfway through the dedicated time period. There are a variety of factors that play a role in scheduling these sessions, such as available faculty/near-peer tutors, number of ultrasound machines, and available physical space.

While full curricula exist for the incorporation of ultrasound into the medical school curriculum, limitations include lack of financial resources and faculty availability with the current curricula focused on faculty-led initiatives [17, 24–29]. Surveys of U.S. medical schools indicated that limited funding and equipment, as well as faculty time, are often barriers to the development of ultrasound curricula in undergraduate medical education [26–28]. Consequently, institutions that do not receive significant national funding or have limited medical education clinical staff have increased difficulty implementing an ultrasound curriculum. The program described in this manuscript was managed by a single EM faculty member during existing paid administration time. To address faculty limitations for the development of this ultrasound program, the M1 ultrasound sessions were organized by M2 students and instructors for the sessions included EM residents, M4 students, and M2 near-peer tutors. Implementing a similar educational program at another institution cannot be a one-size-fits-all approach and will require careful consideration of each institution’s particular resources. To increase flexibility in the integration of ultrasound into existing curricula, these sessions can be broken down into shorter single organ sessions or combined into longer sessions covering more anatomy. Another option would be for all didactic material to be administered in a flipped classroom fashion so that all in-person curricular time is used for hands-on scanning.

The main limitation to our study is the lack of long-term outcomes. Specifically, whether ultrasound sessions improved knowledge long-term and resulted in better test performance and graduates who were better prepared for residency. Additionally, we describe the addition of ultrasound to the undergraduate medical curriculum at a single institution; each institution has its own unique challenges that might not be addressed by our study.

## Conclusions

The are many barriers an institution may face when attempting to incorporate ultrasound education into the undergraduate medical curriculum. In this manuscript, we illustrate a stepwise approach to incorporating ultrasound into undergraduate medical education in a setting that lacks faculty and curricular time. Students found these sessions relevant and informative and felt ultrasound was a useful tool for reinforcing basic science concepts. These results suggest that the next step at our institution should be adding the first-year medical student ultrasound sessions to the formal curriculum.

## Electronic supplementary material

Below is the link to the electronic supplementary material.

Supplementary Material 1

Supplementary Material 2

Supplementary Material 3

Supplementary Material 4

## Data Availability

The datasets used and/or analyzed during the current study are available from the corresponding author on reasonable request.
