# Supplementary material for: Incorporating ultrasound training into undergraduate medical education in a faculty-limited setting
Source: BMC Med Educ. 2023 Apr 19;23:263. doi: 10.1186/s12909-023-04227-y (PMC10113991; doi:10.1186/s12909-023-04227-y)
Supplement: Supplementary file 3 — Supplementary Material 3 [file 12909_2023_4227_MOESM3_ESM.docx]

Abdominal Ultrasound Session Pretest

1. Which is not one of the four major anatomical areas examined during FAST?
   1. Morison’s Pouch
   2. Perisplenic view
   3. Subxiphoid pericardial window
   4. Retroperitoneal recess
   5. Suprapubic window (pouch of Douglas)
2. Extended FAST (e-FAST) includes what additional view(s)?
   1. Bilateral Hemithoraces
   2. Bilateral upper anterior chest wall
   3. Abdominal Aorta
   4. A and B
   5. All of the above
3. Which of the following is not an indication of abdominal ultrasound?
   1. Concern for intra-abdominal blood loss from another source
   2. Trauma
   3. Atypical right-sided chest pain or shoulder pain
   4. Constipation for extended period of time
4. Select the best ultrasound probe to use for an abdominal ultrasound.
   1. Low-frequency curvilinear probe
   2. High-frequency linear probe
   3. Phased array probe

Label this right upper quadrant image:


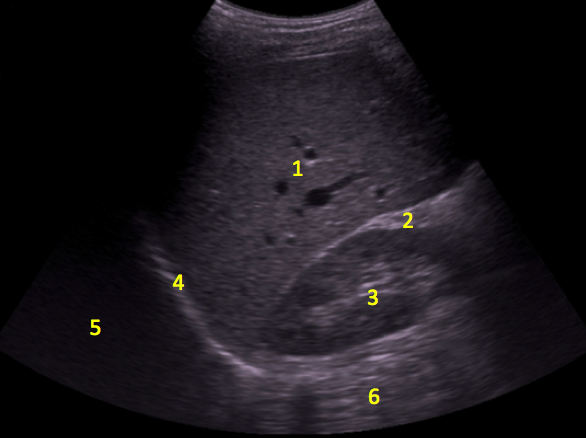


1. ___________________________________________________________
2. ___________________________________________________________
3. ___________________________________________________________
4. ___________________________________________________________
5. ___________________________________________________________
6. ___________________________________________________________

Label this image:


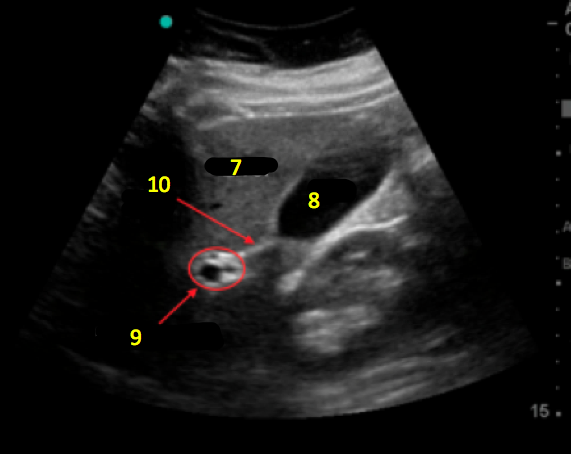


7. _______________________________________________________________

8. _______________________________________________________________

9. _______________________________________________________________

10. _______________________________________________________________
